# Uterine miR-877-3p and let-7a-5p are increased during simulated menstruation in a mouse model

**DOI:** 10.1530/RAF-21-0112

**Published:** 2022-02-17

**Authors:** Marianne Watters, Catherine A Walker, Alison A Murray, Moira Nicol, Jacqueline A Maybin

**Affiliations:** 1 MRC Centre for Reproductive Health, University of Edinburgh, Edinburgh, UK

**Keywords:** hypoxia, menses, miRNA, uterus, endometrium

## Abstract

Heavy periods are common and debilitating, but we do not fully understand how they are caused. Increased understanding of menstrual bleeding could result in new treatments for problematic periods. Low oxygen levels are present in the womb lining during a period. These low oxygen levels help trigger the repair process required to stop menstrual bleeding. MicroRNAs (miRNAs) are small molecules that can affect cell function, and some are regulated by oxygen levels. We examined whether such miRNAs were present in the womb lining during a period. To overcome the variability present in humans, we studied the womb of mice given hormones to mimic the human menstrual cycle. We revealed that two miRNAs known to be regulated by oxygen levels were increased in the womb during menstruation. These miRNAs may help regulate menstrual blood loss and merit further study as a potential target for future treatments for heavy periods.

## Research Letter

Menstrual disorders are common and debilitating. To identify new therapeutic targets, the physiology of menstruation must be delineated. Progesterone withdrawal during the late secretory phase of the menstrual cycle results in endometrial tissue inflammation, hypoxia, and shedding of the luminal portion during menses (Critchley*et al.* 2020). The mouse model of simulated menses has been shown to recapitulate the events of human menstruation, and we have previously detected intense endometrial hypoxia during endometrial shedding in this model (Brasted*et al.* 2003, Maybin*et al.* 2018).

MicroRNAs(miRNAs) are small non-protein-coding molecules that are involved in post-transcriptional gene expression. They play key roles in the regulation of cell differentiation, proliferation, death, and metabolism (Bartel 2004). Hypoxia has been shown to regulate miRNA concentrations in other tissue sites (Kulshreshtha*et al.* 2007), but their presence in the endometrium during menstruation remains undefined. We hypothesised that hypoxia-regulated miRNAs would be increased at menstruation in our mouse model of simulated menses.

Ovariectomised C57BL/6J mice were administered sequential oestradiol and progesterone before induction of decidualisation via transcervical oil injection. Uterine tissue was collected prior to progesterone withdrawal (T0, secretory phase equivalent, *n*  = 6) and 8 h after progesterone withdrawal (T8, menstrual phase equivalent, *n*  = 8). Previous studies have confirmed endometrial breakdown, intense endometrial hypoxia, and vaginal bleeding at T8 in this model (Maybin*et al.* 2018). All experimental animal procedures were approved by the University of Edinburgh ethical committee and performed in accordance with the Animals Scientific Procedures Act (1986) of the UK Home Office (PPL 70/8754).

**Figure 1:**
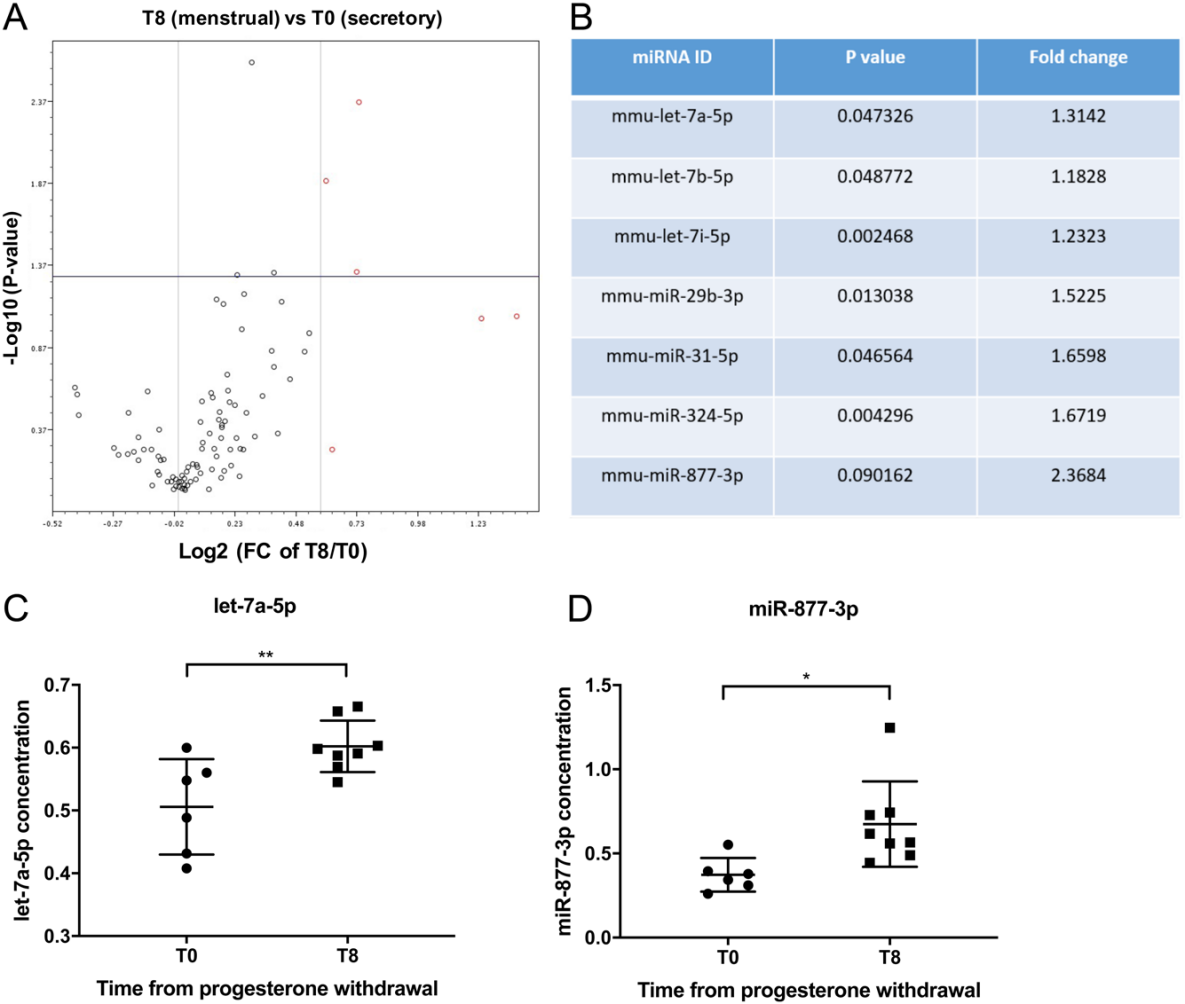
Hypoxia-regulated miRNA in mouse uterine tissue from T0 (prior to progesterone withdrawal; secretory equivalent) and T8 (8 h following progesterone withdrawal; menstrual equivalent and time of endometrial hypoxia). (A) A hypoxia-targeted Qiagen miScript® miRNA PCR array examined 84 hypoxia pathway-related miRNAs, and analysis was carried out using Qiagen Assay Data Analysis Software. Results are displayed in a volcano plot. miRNAs shown in red have the greatest fold change when comparing T8 to T0. Circles above the horizontal line represent statistically significant changes. Reported *P*  values were calculated based on a Student’s *t*-test for each gene (parametric, unpaired, two-sample equal variance, two-tailed distribution). (B) Seven miRNA candidates were selected for validation; all six displayed significant changes plus one candidate with the largest fold change. Of these seven candidates, let-7a-5p (C) and miR-877-3p (D) showed significantly increased concentrations (unpaired Student’s *t*-test) in uterine tissue from T8 vs T0 when examined by RT-qPCR. FC: fold change, *P < .05, **P < .01, horizontal lines: mean, error bars: standard deviation.

A hypoxia pathway-focused miScript® miRNA PCR array was used for miRNA expression profiling of a subset of mouse uterine tissue collected at T0 (*n* = 4) and T8 (*n* = 4). Of 84 hypoxia pathway-related miRNAs tested, 7 candidate miRNAs were selected, the 6 transcripts with significant fold changes and 1 transcript with the greatest fold change between T0 (‘secretory’) and T8 (‘menstrual’) (Fig. 1A and B). Two of the seven identified miRNA candidates were validated by RT-qPCR of all available uterine tissue, revealing significantly increased concentrations of let-7a-5p and miR-877-3p in uterine tissue collected at T8 (menstrual, *n*  = 8) vs T0 (secretory, *n*  = 6) (Fig. 1C and D).

A physiological hypoxic episode occurs transiently in the endometrium of this mouse model during simulated menstruation (Maybin*et al.* 2018). We detected increased concentrations of let-7a-5p and miR-877-3p in uterine tissue collected 8 h following progesterone withdrawal (i.e. in tissue exposed to *in vivo* hypoxia) vs that collected when progesterone levels remained high.

It remains to be determined whether these miRNAs are regulated by hypoxia, progesterone withdrawal, inflammation or another mechanism. In addition, their levels in the human menstrual endometrium are unknown. The role of let-7a-5p and miR-877-3p in the endometrium is undefined. In cancer, let-7a-5p has been shown to inhibit migration, invasion and epithelial–mesenchymal transition, and miR-877-3p promoted cell proliferation and differentiation (Duan*et al.* 2019, Mendaza*et al.* 2021). We postulate that these miRNAs fine-tune the endometrial transcriptional response to menstrual hypoxia to ensure timely repair of the denuded endometrial surface and limit menstrual blood loss. Further examination of the regulation and function of endometrial miRNAs at menstruation is warranted and may result in increased understanding of menstrual physiology and identification of novel therapeutic targets for those with problematic menstrual bleeding.

## Declaration of interest

The authors declare that there is no conflict of interest that could be perceived as prejudicing the impartiality of the research letter.

## Funding

This work was funded by a Tenovus http://dx.doi.org/10.13039/501100000723 Scotland Research Grant, Wellbeing of Women http://dx.doi.org/10.13039/501100000325 grant RG1820 and Wellcome Trust http://dx.doi.org/10.13039/100010269 Grant 209589/Z/17/Z. The experiments described herein were undertaken in the MRC Centre for Reproductive Health which is funded by MRC Centre Grants G1002033 and MR/N022556/1.

## Author contribution statement

J A M, A M and C A W designed the study. M W, C A W, A M and M N performed the experiments and analysed the data. M W and J A M wrote the paper. All authors edited and approved the manuscript.
